# Decreased renal perfusion during acute kidney injury in critical COVID-19 assessed by magnetic resonance imaging: a prospective case control study

**DOI:** 10.1186/s13054-022-04132-8

**Published:** 2022-09-01

**Authors:** Tomas Luther, Per Eckerbom, Eleanor Cox, Miklos Lipcsey, Sara Bülow, Michael Hultström, Francisco Martinez Torrente, Jan Weis, Fredrik Palm, Susan Francis, Robert Frithiof, Per Liss

**Affiliations:** 1 Anaesthesiology and Intensive Care Medicine, Department of Surgical Sciences, Uppsala University Hospital, Uppsala University, 751 85 Uppsala, Sweden; 2 Section of Radiology, Department of Surgical Sciences, Uppsala University, Uppsala, Sweden; 3 Sir Peter Mansfield Imaging Centre, School of Physics & Astronomy, University of Nottingham, Nottingham, UK; 4 NIHR Nottingham Biomedical Research Centre, Nottingham University Hospitals NHS Trust and the University of Nottingham, Nottingham, UK; 5 Hedenstierna Laboratory, Department of Surgical Sciences, Uppsala University, Uppsala, Sweden; 6 Integrative Physiology, Department Medical Cell Biology, Uppsala University, Uppsala, Sweden; 7 Department of Medical Physics, Uppsala University Hospital, Uppsala, Sweden

## Abstract

**Background:**

Renal hypoperfusion has been suggested to contribute to the development of acute kidney injury (AKI) in critical COVID-19. However, limited data exist to support this. We aim to investigate the differences in renal perfusion, oxygenation and water diffusion using multiparametric magnetic resonance imaging in critically ill COVID-19 patients with and without AKI.

**Methods:**

A prospective case–control study where patients without prior kidney disease treated in intensive care for respiratory failure due to COVID-19 were examined. Kidney Disease: Improving Global Outcomes Creatinine criteria were used for group allocation. Main comparisons were tested using Mann–Whitney U test.

**Results:**

Nineteen patients were examined, ten with AKI and nine without AKI. Patients with AKI were examined in median 1 [0–2] day after criteria fulfillment. Age and baseline Plasma-Creatinine were similar in both groups. Total renal blood flow was lower in patients with AKI compared with patients without (median 645 quartile range [423–753] vs. 859 [746–920] ml/min, *p* = 0.037). Regional perfusion was reduced in both cortex (76 [51–112] vs. 146 [123–169] ml/100 g/min, *p* = 0.015) and medulla (28 [18–47] vs. 47 [38–73] ml/100 g/min, *p* = 0.03). Renal venous saturation was similar in both groups (72% [64–75] vs. 72% [63–84], ns.), as was regional oxygenation (*R*_2_*) in cortex (17 [16–19] vs. 17 [16–18] 1/s, ns.) and medulla (29 [24–39] vs. 27 [23–29] 1/s, ns.).

**Conclusions:**

In critically ill COVID-19 patients with AKI, the total, cortical and medullary renal blood flows were reduced compared with similar patients without AKI, whereas no differences in renal oxygenation were demonstrable in this setting.

*Trial registration* ClinicalTrials ID: NCT02765191, registered May 6 2014 and updated May 7 2020.

**Graphic Abstract:**

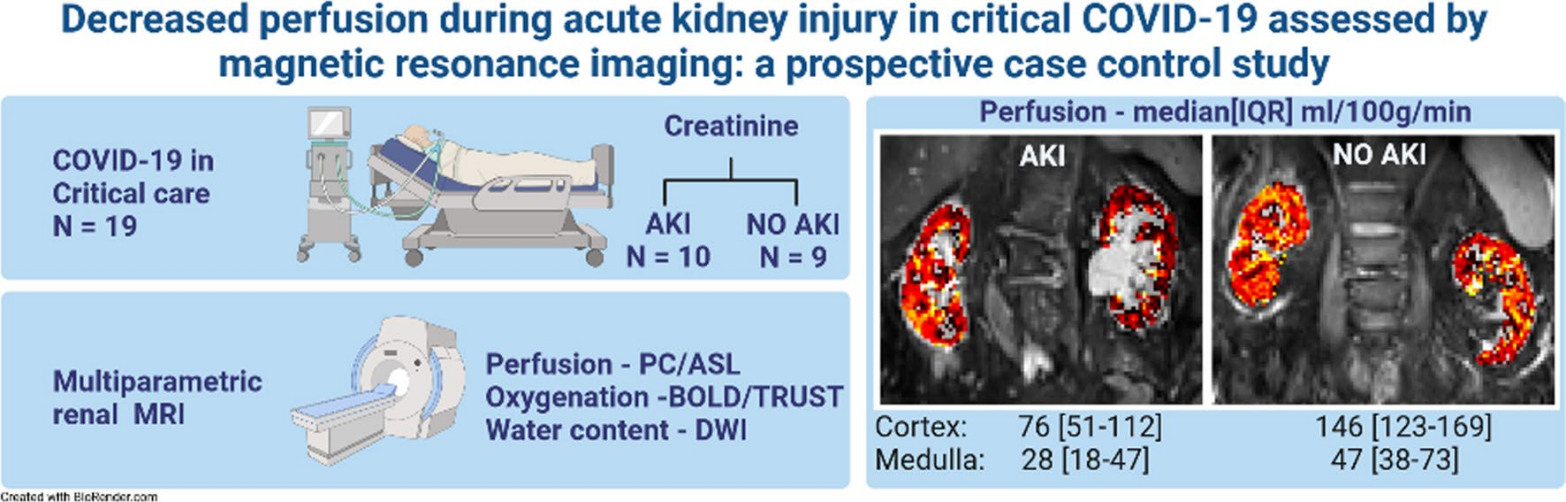

**Supplementary Information:**

The online version contains supplementary material available at 10.1186/s13054-022-04132-8.

## Introduction

Acute kidney injury (AKI) is independently associated with increased mortality in hospitalized patients with corona virus disease 19 (COVID-19) and may result in higher odds of death than AKI due to other causes [1]. AKI also increases the risk of impaired kidney function in surviving patients after the acute phase of COVID-19 [2]. A recent meta-analysis of patients in intensive care units (ICUs) from several continents estimated an incidence of AKI of 46%, with 19% receiving renal replacement therapy (RRT) [3].

The pathogenesis of AKI in COVID-19 has several contributing factors [4]. Since the majority of critically ill COVID-19 patients who develop AKI do so within 24 h of intubation [5], altered renal hemodynamics as a consequence of the application of a positive end-expiratory pressure (PEEP) and the administration of sedative drugs with cardiovascular depressing effects may contribute to the development of AKI in critically ill patients with COVID-19. This is consistent with the early occurrence of severe oliguria previously reported by our group [6]. Reduced renal perfusion and increased renovascular resistance have been demonstrated in AKI due to non-infectious causes as well as AKI associated to sepsis [7–9]. However, whether these are causative or a secondary feature of AKI is still unknown. An inability to reduce kidney oxygen consumption by limiting tubular transport of sodium has been associated with AKI [7, 10], which in combination with reduced perfusion and oxygen delivery result in renal hypoxia. Emerging non-invasive multiparametric magnetic resonance imaging (mpMRI) techniques offer novel possibilities to investigate perfusion, oxygenation and tissue characteristics in kidney disease [11–13].

We hypothesize that AKI development during critical COVID-19 is associated with reduced renal blood flow, impaired renal oxygenation and increased renal water content. Here, we aim to investigate differences in perfusion, oxygenation and water diffusion using MRI in critically ill COVID-19 patients with or without AKI.

## Material and methods

### Patient cohort and study design

The study was approved by the Uppsala Regional Ethical Review Agency (No. 2014/381 with amendment No. 2020-01996 and No. 2021-04798). Informed consent was obtained from each patient, or next of kin if the patient was unable to give consent. The Declaration of Helsinki and its subsequent revisions were followed. This is a prospective case control sub-study of the MR-Evaluation of Renal Function In Septic Patients (MERSEP) study, the protocol of the study was pre-registered (ClinicalTrials ID: NCT02765191), first registered in May 6 2014 with a COVID-19 updated protocol registered May 7 2020 prior to the first patient being enrolled. The study was conducted at Uppsala University Hospital, a tertiary care center in Uppsala, Sweden. The main end-point comparisons were predefined as between-group differences of the measures included in renal mpMRI between patients with AKI or no/low grade AKI (AKI group and NO AKI group). All recruited patients that completed at least one scan session are included in this paper. Due to the novelty of the mpMRI, healthy volunteer data provided from an existing cohort collected at the Sir Peter Mansfield Imaging Centre, Nottingham, UK with identical mpMRI sequences (approved by the Faculty of Medicine and Health Sciences Research Ethics Committee E14032013), have been added post hoc for secondary comparison of measurements of perfusion, oxygenation, and *T*_1_ to facilitate interpretation.

Adult patients with polymerase chain reaction (PCR) confirmed COVID-19 and AKI or at risk of AKI development admitted to the ICU were screened for inclusion. Exclusion criteria were pregnancy, preexisting end stage renal failure or dialysis, contraindications for MRI-scanning (e.g., pacemaker or certain metal implants), deterioration or instability in vital parameters to a degree where MRI is not feasible (e.g., dependence of prone-positioning). Group participation in the AKI group was determined based on the Kidney Disease Improving Global Outcome (KDIGO) creatinine criteria only [14] due to common occurrence of oliguria without a reduction in glomerular filtration [6]. Baseline Plasma (P)-Creatinine was determined as the lowest value within a normal range during the previous 6 months up to MRI examination. Group allocation to the AKI group was determined as fulfillment of the KDIGO Creatinine criteria at the day of the MRI examination, or within twelve hours after the MRI examination. All other patients were assigned as NO AKI. Measurement of P-Creatinine was made at least every morning during ICU stay. Group sizes of *n* = 10 were calculated to have statistical power (1 − *β*) of ≥ 0.8 and alpha coefficient ≤ 0.05 for a 20% difference in total renal blood flow and 10% in oxygenation using data from healthy volunteers [11].

Patients were transported to the MRI scanner by dedicated ICU-staff. Mechanically ventilated patients were ventilated with a Maquet Servo-i MR-Conditional ventilator (Getinge AB, Sweden) during the MRI examination with the same positive end-expiratory pressure (PEEP) as before transport and fraction of inhaled oxygen (FiO_2_), respiratory frequency and inspiratory pressures adjusted to maintain target blood oxygen saturation (SpO_2_) and minute ventilation. Sedation regime and vasoactive treatment, when present, was continued throughout the MRI examination. Saturation with pulse oximetry and invasive arterial pressure was monitored continuously and recorded manually every 5 min. Remaining medical data and history were collected from the patients’ electronic medical record. Laboratory investigations were performed by the Department of Clinical Chemistry as in clinical practice.

Details of the renal multiparametric MRI measures have been described in a previous publication and are summarized regarding technique and output parameter in Table 1 [15]. Participants were scanned on a 3T MR scanner (Achieva dStream, Philips Healthcare, Best, The Netherlands) in a supine position. The MRI protocol was designed to be ~ 35–40 min in duration, with MRI parameters guided by previous studies [15–17]. A full description of the MRI acquisition and analysis can be found in Additional file 1. MRI data analysis was performed blinded to AKI status. Healthy volunteers’ data were taken from a previously published study [16], performed on the same field strength and vendor MR scanner (Philips 3T Achieva) using identical pulse sequence parameters.

**Table 1:** Description of multiparametric renal MRI measures

Name:	Measurement of blood flow in renal arteries and veins. Sensitized to flow by using bipolar gradients affecting the phase signal of spins that flow with a uniform velocity in the direction parallel to the gradients. By utilizing ECG gating, blood velocity and vessel area are measured across 15–20 points in the cardiac cycle during a breath hold of approximately 15–20 s. Global perfusion of the kidney can be measured by dividing total blood flow to the kidney by the total kidney volume (TKV). Causes of systematic falsely low estimations of blood flow include: non-perpendicular placement of the imaging plane, inaccurate estimation of elastic dilatation of the artery during cardiac cycle, aberrant arteries or placement of field down stream of arterial bifurcations. Intra-individual coefficient of variation is quoted to be 14%
Phase Contrast (PC)	
Category:	
Global perfusion	
Output:	
Total renal blood flow (ml/min)	
Name:	A subtraction technique where arterial blood water is labelled (inverted) prior to imaging. Difference signals are determined by subtracting imaging data with and without labeling. Data are collected using respiratory triggering. The resulting ASL difference images are dependent on tissue perfusion, with regional perfusion in the cortex and medulla calculated from a kinetic model. A Gaussian fit to all voxels within the perfusion maps in cortex and medulla masks is performed. Edema may introduce bias as blood/tissue coefficient is assumed constant. Intra-individual coefficient of variation is 9%
Arterial Spin Labeling (ASL)	
Category:	
Regional perfusion	
Output:	
Regional perfusion Cortex and Medulla (ml/100 g/min)	
Name:	Deoxyhemoglobin is paramagnetic and shortens the transverse relaxation constant *T*_2_* (ms) which is the inverse of the relaxation rate *R*_2_* (1/s). Images are collected during a breath hold of approximately 15–20 s. Besides oxygenation, *R*_2_* is also influenced by changes in hematocrit and tissue water content. Increased water content prolongs both *T*_2_ and *T*_2_* (and shortens *R*_2_*) relaxation times. Intra-individual coefficient of variation in *R*_2_* is 4%
Blood Oxygen Level Dependent (BOLD)	
Category:	
Regional oxygenation	
Output:	
*R*_2_* (relative measure of oxygenation)	
Name:	Spin tagging of blood, similar to ASL, is used to separate the signals from venous blood from surrounding tissues, and this is collected across a range of *T*_2_-weighted echo times. By acquiring an *R*_2_ signal solely from venous blood, the venous oxygenation (saturation) can be calculated. In contrast to BOLD, TRUST data are not influenced by edema and hematocrit. Renal TRUST is a novel technique with limited previous data and is less explored compared with BOLD. Validation studies are mainly from the central nervous system to study the sagittal sinus
*T*_2_ Relaxation Under Spin Tagging (TRUST)	
Category:	
Global oxygenation	
Output:	
Renal venous saturation (%)	
Name:	DWI determines signals from the Brownian motion of water in tissue by acquiring data at a range of b-values which alters the measured apparent diffusion coefficient (ADC). ADC is increased in the presence of edema. Incorporation of the IntraVoxel Incoherent Motion (IVIM) bi-exponential model is used to calculate the pure diffusion of water in tissue coefficient (*D*) separated from pseudodiffusion (D*) representing microscopic intravoxel flows of blood or urine, and the perfusion fraction *f*_*p*_ (%). Intra-individual coefficient of variation in ADC is typically 3%, while those of *D*, D* and *f*_*p*_ are 9, 39, 22%
Diffusion weighted imaging (DWI)	
Category:	
Regional water diffusion	
Output:	
Apparent diffusion coefficient (ADC), *D*, D*, *f*_*p*_	
Name:	Structural imaging and relaxation time mapping. Signal intensity and contrast between tissues can be manipulated by repetition time and echo time of the measurement sequences. A strongly *T*2-weighted sequence allows total kidney volume (TKV) to be measured. Absolute values of tissue relaxation times differ between 1.5T and 3T scanners. *T*_1_ mapping in this study is performed using a respiratory triggering inversion recovery technique, with a curve fitting function used to obtain a *T*_1_ value. *T*_2_ mapping is performed using a respiratory triggered GRASE scheme. Intra-individual coefficient of variation in *T*_1_-mapping is 2%, whereas intra-individual coefficient of variation in TKV is 4%
*T*_2_-weighted imaging, and *T*_1_ and *T*_2_ mapping	
Category:	
Structure	
Output:	
Total kidney volume (TKV), *T*_1_ and *T*_2_ relaxation times	

Outline of multiparametric renal MRI measures collected in the study divided into different categories, as detailed in a previous published description [15]

### Statistical analysis

Continuous variables are expressed as median [interquartile range]. The mean of the measured variable from both kidneys was used as the end point for comparison. If a measurement in a single kidney was missing or unreliable, the value from the other kidney was used instead. Missing data were otherwise excluded. Kruskal–Wallis one-way analysis of variance was used to compare the two study groups with healthy volunteers. Between-group differences of continuous variables were tested using a Mann–Whitney U test. Correlations between continuous variables were calculated using Product Moment Correlation (Pearson) in GraphPad Prism (version 9.3.1 for Windows, GraphPad Software, San Diego, California USA, www.graphpad.com). Descriptive data were calculated using Excel 2016 (Microsoft, Santa Rosa, California), and other statistical calculations were made using Statistica 13.5.0.17 (TIBCO Software, Palo Alto California). Graphs were made using SigmaPlot 14.0 (Systat Software, San Jose, California) and MATLAB (The MathWorks, Inc).

## Results

### Patient cohort

Nineteen (19) patients treated in ICU for acute respiratory failure due to COVID-19 were included in the study. The median age of patients was 65 [61–72] years, comparable to the healthy volunteer groups’ median age of 65 [58–73] years. Comorbidities were common in the study cohort with COVID-19. There was a history of hypertension in 63% of patients, 32% had diabetes mellitus and 68% were treated with angiotensin converting enzyme inhibitor (ACEi) or angiotensin receptor blocker (ARB) before hospital admission. Dexamethasone was used to treat 79% of patients to improve patient outcome in COVID-19 with similar proportions between the two groups (Table 2). Acute respiratory distress syndrome (ARDS) of at least moderate severity was diagnosed during the ICU stay in 95% of the patients including all patients in the AKI group.

**Table 2:** Patient characteristics, comorbidities and outcome

	AKI (*N* = 10)	NO AKI (*N* = 9)
Age, years [IQR]	66 [64–72]	65 [53–70]
Male, *n* (%)	8 (80%)	8 (89%)
Height, cm [IQR]	173 [169–177]	180 [170–187]
Weight, kg [IQR]	94 [82–102]	86 [80–102]
Body Mass Index, [IQR]	32 [27–35]	27 [26–36]
Hypertension, *n* (%)	7 (70%)	5 (56%)
History of treatment with ARB/ACEi, *n* (%)	7 (70%)	6 (67%)
Diabetes, *n* (%)	4 (40%)	2 (22%)
Ischemic heart disease or congestive heart failure, *n* (%)	3 (30%)	2 (22%)
Ischemic heart disease, *n* (%)	3 (30%)	1 (11%)
Congestive heart failure, *n* (%)	0 (0%)	1 (11%)
Asthma or COPD, *n* (%)	2 (20%)	0 (0%)
History of CKD, *n* (%)	0 (0%)	0 (0%)
Baseline Plasma-Creatinine, µmol/l [IQR]	68 [65–77]	66 [58–73]
Most severe AKI stage during hospital stay, [IQR]	2 [2, 3]	0[0–1]
AKI stage 2 or 3 any time during hospital stay, *n* (%)	8 (80%)	0 (0%)
RRT at any time in ICU, *n* (%)	2 (20%)	0 (0%)
SAPS 3, [IQR]	54 [52–56]	53 [50–55]
Days of symptomatic COVID-19 at ICU-admission, [IQR]	9 [8–11]	9 [9, 10]
Treatment with dexametasone, *n* (%)	8 (80%)	7 (78%)
IMV at any time during ICU stay, *n* (%)	10 (100%)	7 (78%)
Days with IMV, [IQR]	18 [15–22]	14 [6–18]
Vasoactive treatment at any time in ICU, *n* (%)	10 (100%)	7 (78%)
Moderate or severe ARDS, *n* (%)	10 (100%)	8 (89%)
Severe ARDS, *n* (%)	8 (80%)	5 (56%)
90-day survival, *n* (%)	6 (60%)	6 (67%)

Patient characteristics, comorbidities and outcome in the 19 patients treated in ICU for respiratory failure due to COVID-19 included in study

*IQR* interquartile range, *ARB* angiotensin II receptor blocking drug, *ACEi* angiotensin converting enzyme inhibitor, *COPD* chronic obstructive pulmonary disease, *CKD* chronic kidney disease, *AKI* acute kidney injury, *RRT* renal replacement therapy, *ICU* intensive care unit, *SAPS 3* Simplified Acute Physiology Score 3, *IMV* invasive mechanical ventilation, *ARDS* acute respiratory distress syndrome

All patients had at least one measurement of P-Creatinine within normal range during the current hospitalization prior to the MRI examination. In the NO AKI group, none of the patients fulfilled KDIGO Creatinine criteria during the first 48 h following the MRI examination. During the following ICU-care, two patients in the AKI group received renal replacement therapy (RRT). During the whole course of hospitalization, 80% of patients in the AKI group and none (0%) in the NO AKI group had at least one episode of severe AKI (Stage 2 or 3 according to KDIGO creatinine criteria). At 90 days from inclusion, 12 patients were still alive. Patient characteristics, comorbidities and outcomes are further presented groupwise in Table 2.

At the time of the MRI examination (Table 3), the patients had been treated in the ICU for 4 [3–8] days of which 89% required invasive ventilation for the previous 3 [2–4] days. In the AKI group, the KDIGO Creatinine criteria for current episode of AKI were fulfilled in median 1 day [0–2] prior to the examination and two (20%) of patients already had developed severe AKI, stage 2 or 3 at the time of the examination. The patients were general circulatory stable. Of the 63% of patients receiving vasoactive drugs, low doses were used, one received 2 µg/kg/min of dobutamine and the others received no more than 0.1 µg/kg/min noradrenaline. All patients had a mean arterial pressure above 65 mmHg and all but two patients had a P-Lactate below 2 mmol/l. There were no adverse events recorded during the examinations.

**Table 3:** Patient characteristics of physiological parameters at MRI examination

	AKI (*N* = 10)	NO AKI (*N* = 9)
Current Plasma-Creatinine, µmol/l [IQR]	104 [101–114]	67 [64–77]
Current eGFR (Creatinine), ml/min [IQR]	56 [49–59]	87 [78–90]
Current KDIGO Creatinine AKI Stage	1 [1–1]	
Last recorded hourly urine output, ml/kg [IQR]	0.9 [0.3–1.7]	1 [0.8–1.7]
Last recorded hourly urine output, ml [IQR]	65 [35–131]	110 [80–150]
Furosemide within 12 h before MRI scan, mg [IQR]	8 [1–10]	0 [0–0]
Time since latest furosemide, h [IQR]	7 [5–20]	28 [17–36]
Any furosemide within 3 h before MRI scan, *n* (%)	2 (20%)	1 (11%)
Net fluid intake at examination day, ml [IQR]	318 [− 15 to 629]	184 [-16–429]
SOFA score, points [IQR]	7 [7, 8]	6 [4–6]
Days of symptomatic COVID-19, *n* [IQR]	17 [14–19]	12 [11–14]
Days in ICU at examination, *n* [IQR]	8 [4–8]	3 [2–5]
Plasma-CRP, mg/l [IQR]	98 [71–140]	115 [64–186]
Days since start of invasive ventilation, *n* [IQR]	4 [2–5]	2 [2, 3]
Arterial oxygen saturation	96% [93–97]	95% [95–98]
Arterial pO_2_, kPa [IQR]	10 [10, 11]	10 [10–12]
Arterial pCO_2_, kPa [IQR]	6.3 [5.7–6.6]	5.5 [5.2–6]
Arterial pH, [IQR]	7.39 [7.37–7.41]	7.42 [7.4–7.44]
P/f-ratio, kPa [IQR]	21 [20–27]	26 [25–33]
PEEP, cmH_2_O [IQR]	14 [13–15]	10 [9–12]
Mean arterial pressure, mmHg [IQR]	80 [79–85]	81 [80–98]
Sinus rhythm, *n* (%)	10 (100%)	8 (89%)
Blood hemoglobin, g/dl [IQR]	11.5 [11.3–12.3]	12.8 [11.4–12.9]
Central venous saturation, [IQR]	73% [72–77]	67% [65–74]
Vasoactive drug, *n* (%)	8 (80%)	4 (44%)
Noradrenaline dose, µg/kg/min [IQR]	0.05 [0.01–0.07]	0 [0–0.03]
Plasma-lactate, mmol/l [IQR]	1.4 [1–1.9]	1.5 [1.3–1.6]
Plasma-NT-proBNP, ng/l [IQR]	201 [131–633]	373 [236–491]

Patient characteristics of physiological parameters on the day of the MRI examination in the 19 patients included in the study who were treated in ICU for respiratory failure due to COVID-19

*IQR* interquartile range, *eGFR* estimated glomerular filtration rate, *SOFA* Sequential Organ Failure Assessment, *CRP* C-reactive protein, *P/f* PaO_2_/FiO_2_, *PEEP* positive end-expiratory pressure, *NT-proBNP* N-terminal pro-brain natriuretic peptide

### Multiparametric MRI

**Fig. 1:**
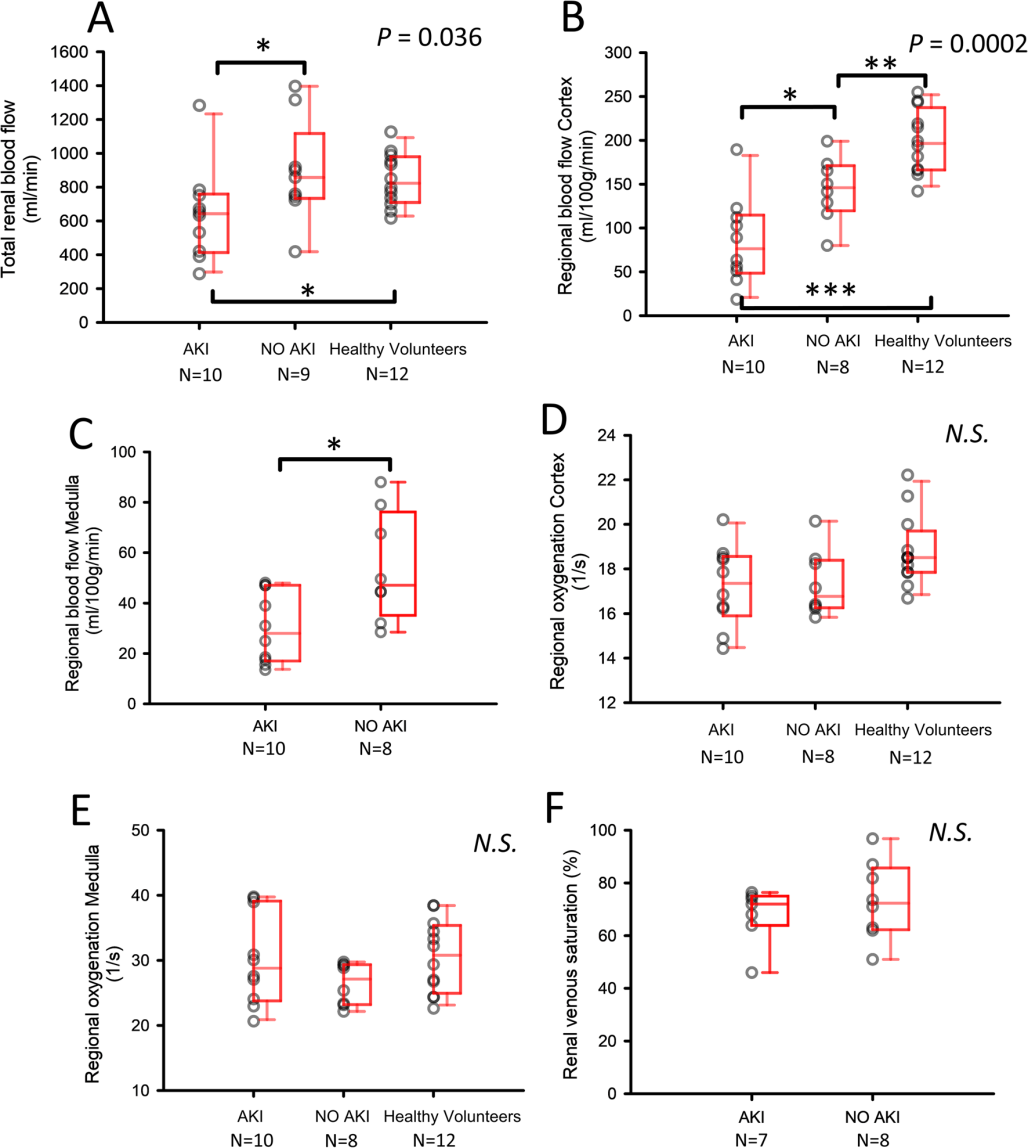
Main results of renal multiparametric MRI. Box- and scatterplots of renal multiparametric MRI in 19 patients treated in ICU for respiratory failure due to COVID-19 with AKI and NO AKI, along with 12 healthy volunteers of similar age. Valid numbers of MRI examinations are specified for each parameter and group. *p* values from Kruskal–Wallis ANOVA. *, **, *** signifies *p* < 0.05, 0.01 and 0.001 in Mann–Whitney U test. TRUST is short for *T*_2_ Relaxation Under Spin Tagging

Not all parameters could be obtained in all subjects due to technical issues related to the scanner or significant artifacts within the data. The number of valid examinations is specified for each mpMRI measure in Fig. 1 and Table 4.

**Table 4:** Additional results of renal multiparametric MRI

	AKI	*n*	NO AKI	*n*	Healthy volunteers	*n*
*Perfusion*						
Global perfusion (ml/100 g/min)	168 [142–216]	10	202 [174–235]	9	220 [174–242]	12
Regional perfusion ratio (cortex/medulla)	2.4 [2.2–3.3]	10	2.2 [1.7–3.4]	7		
Resistance index*	0.90 [0.82–0.93]	10	0.79 [0.75–0.86]	8		
*Structure and regional water diffusion*						
Total kidney volume (ml)	356 [331–437]	10	390 [359–447]	9	403 [345–430]	12
*T*_1_ cortex (ms)†	1560 [1524–1638]	8	1522 [1497–1638]	8	1459 [1400–1525]	12
*T*_1_ medulla (ms)	1838 [1732–1872]	8	1792 [1695–1870]	8	1732 [1661–1850]	12
*T*_2_ cortex (ms)	120 [113–134]	8	124 [119–141]	8		
DWI cortex ADC (× 10^–3^ mm^2^/s)	1.9 [1.9–2.1]	8	2.1 [1.9–2.1]	8		
DWI medulla ADC (× 10^–3^ mm^2^/s)	1.9 [1.9–2.0]	8	2.1 [1.8–2.1]	8		
DWI cortex *D* (× 10^–3^ mm^2^/s)	1.8 [1.7–1.9]	8	1.9 [1.7–2]	8		
DWI cortex D* (× 10^–3^ mm^2^/s)	26 [23–29]	8	27 [24–28]	8		
DWI cortex *f*_*p*_	0.13 [0.11–0.15]	8	0.13 [0.11–0.14]	8		
DWI medulla *D* (× 10^–3^ mm^2^/s)	1.8 [1.7–1.9]	8	1.9 [1.7–2]	8		
DWI medulla D* (× 10^–3^ mm^2^/s)	26 [23–17]	8	27 [26–28]	8		
DWI medulla *F*_P_	0.14 [0.11–0.17]	8	0.15 [0.12–0.16]	8		

Results of renal multiparametric MRI in 19 patients treated in ICU for respiratory failure due to COVID-19 with AKI or NO AKI, and 12 healthy volunteers of similar age. Data presented as median [quartile range]. Significant differences from Mann–Whitney U test between AKI and NO AKI groups are indicated by * if *p* < 0.05. Significant differences from one way Kruskal–Wallis ANOVA between all groups are indicated by † if *p* < 0.05 and ††† if *p* < 0.001. Valid numbers of MRI examinations are specified for each measure and group. Additional results are presented in Fig. 1

*T*_1_ longitudinal relaxation time, *T*_2_ transverse relaxation time, *DWI* diffusion weighted imaging, *ADC* apparent diffusion coefficient, *D* pure diffusion, D* pseudodiffusion, *f*_*p*_ perfusion fraction

### Total renal blood flow measured by phase contrast (Fig. 1a)

Total renal blood flow (RBF) was lower in the AKI group compared with the NO AKI group (645 ml/min [423–753] vs. 859 ml/min [746–920], *p* = 0.037). RBF in the NO AKI group was similar to that in healthy controls, 825 ml/min [720–972] (n.s.). Adjusting RBF by total kidney volume attenuated the differences between groups and rendered them not statistically significant (Table 4). Renal resistive index (RI) could be determined in all but one patient in the NO AKI group and was higher in the AKI group compared with the NO AKI group (0.90 [0.82–0.93] vs. 0.79 [0.75–0.86], *p* < 0.046, Table 4).

### Regional renal tissue perfusion measured by ASL (Fig. 1b, c)

**Fig. 2:**
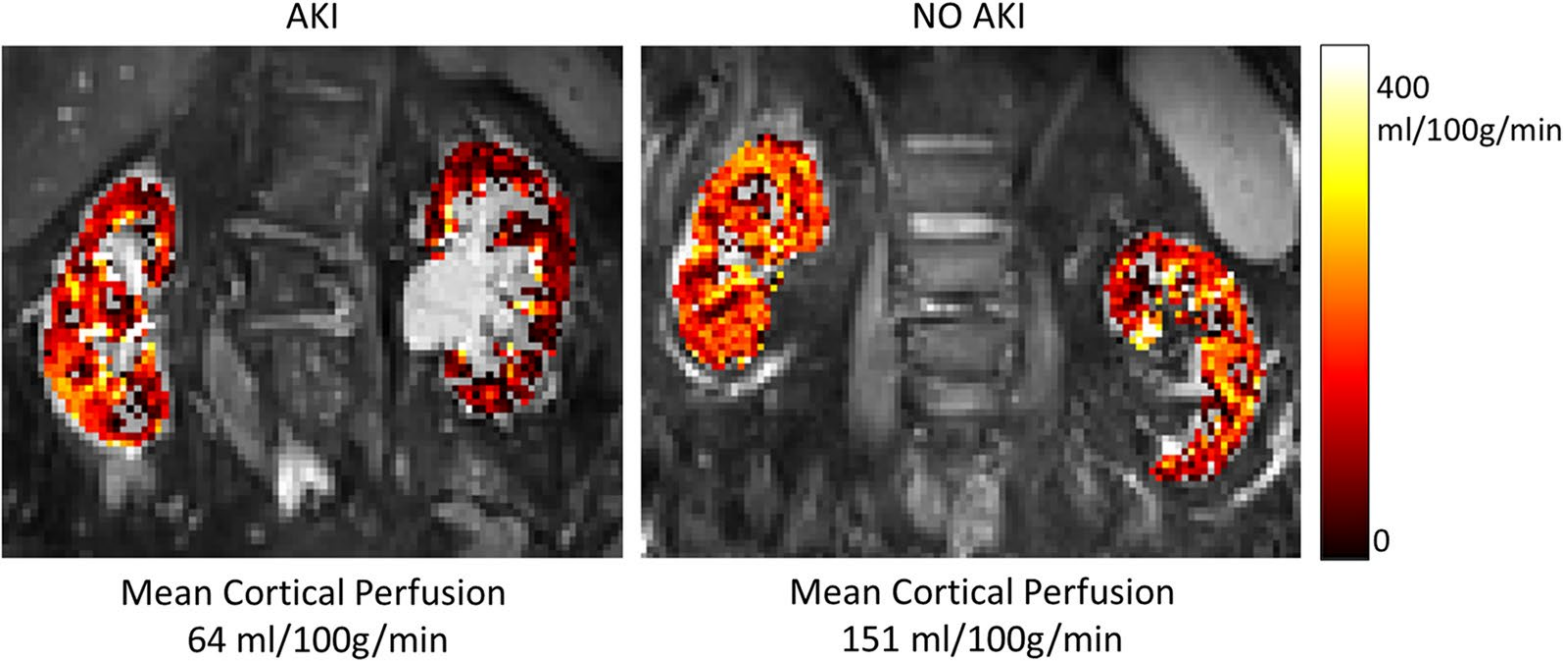
Example of imaging data. Representative regional perfusion using arterial spin labeling with perfusion maps colorized voxelwise showing representative AKI and NO AKI scans from the group of 19 patients with COVID-19 with and without AKI treated in ICU due to respiratory failure. Individual mean cortex perfusion across both kidneys are provided below each image after voxelwise Gaussian fit in each kidney to the histogram of cortical values

There were significant differences in cortical perfusion computed by ASL between the groups (*p* < 0.001). Lowest cortical perfusion was present in the AKI group at 76 ml/100 g/min [51–112], while the NO AKI group had cortical perfusion of 146 ml/100 g/min [123–169] (*p* = 0.015). The cortical perfusion in the NO AKI group was lower compared with healthy volunteers’ 197 ml/100 g/min [167–231] (*p* = 0.009). Medullary perfusion was also reduced in the AKI group compared with the NO AKI group (28 ml/100 g/min [18–47] vs. 47 ml/100 g/min [38–73], *p* = 0.03). There was a similar proportion of regional perfusion (Cortical/Medullary perfusion) in the two patient groups with ratios of 2.4 [2.2–3.3] and 2.2 [1.7–3.4] (n.s.) for the AKI and NO AKI groups, respectively. A representative image of ASL perfusion from each group is presented in Fig. 2.

### Regional and global oxygenation measured by BOLD *R*_2_* and TRUST (Fig. 1d–f)

We could not demonstrate any differences between the groups in either cortical or medullary oxygenation (Fig. 1d, e). Cortical *R*_2_* was 17 (1/s) [16–19] in the AKI group and 17 (1/s) [16–18] in the NO AKI group. In the renal medulla, *R*_2_* was 29 (1/s) [24–39] in patients with AKI compared with 27 (1/s) [23–29] in patients with NO AKI. *R*_2_* values were similar to healthy volunteers’ (Fig. 1d, e). Left renal venous saturation assessed with TRUST was also similar between the AKI and the NO AKI groups (72% [64–75] vs. 72% [63–84], ns.) with large variations within groups (Fig. 1f).

### Regional tissue composition and water diffusion

Cortical and medullary ADC, *D*, D* or *f*_*p*_, and tissue composition (cortical *T*_1_ and *T*_2_ and medullary *T*_1_) did not differ between the AKI and NO AKI groups (Table 4). Cortical *T*_1_ was longer in the AKI group (1560 ms [1524–1638]) compared with healthy volunteers (1459 ms [1400–1525], *p* = 0.009).

### Post hoc analyses

**Fig. 3:**
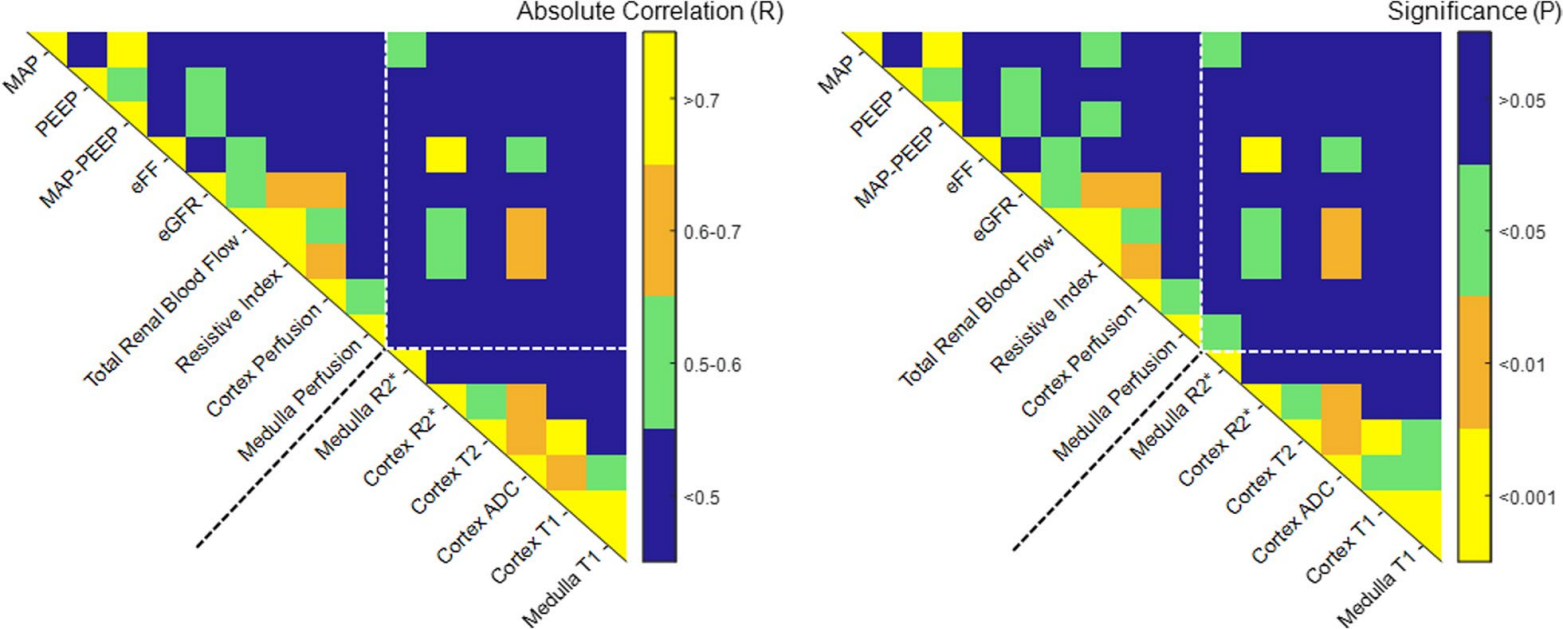
Post hoc correlation matrix. Post hoc correlation matrix for combined patient groups of clinical factors (MAP, eFF and eGFR) and multiparametric MRI measures showing absolute correlation (*R*) and the associated significance (*P*). eFF calculated as eGFR_Creatinine_/(TRBF_Phase Contrast_ × (1 − Hematocrit)). Selected significant correlations of clinical factors and multiparametric MRI measures are shown in Additional file 1. *MAP* mean arterial pressure, *PEEP* positive end-expiratory pressure, *eFF* estimated filtration fraction, *eGFR* estimated glomerular filtration rate, *R*_2_* BOLD relaxation rate, *ADC* apparent diffusion coefficient, *T*_2_ transverse relaxation time, *T*_1_ longitudinal relaxation time, *TRBF* total renal blood flow

Correlations were made post hoc using the entire study population with COVID-19 examining relations between physiological parameters and perfusion, as well as imaging data affected by changes in water content. This was performed to explore physiological factors which may affect renal perfusion and to facilitate the interpretation of the regional oxygenation data. Correlations are summarized in matrices in Fig. 3.

## Discussion

The main findings in this study are that in critically ill COVID-19 patients with AKI, the total, cortical and medullary renal blood flows are reduced compared with patients without AKI, as assessed by magnetic resonance imaging. There were no demonstrable differences in regional or global renal oxygenation, tissue composition or water diffusion. The findings are consistent with the hypothesis that impaired renal blood flow contributes to AKI in COVID-19.

Our observations of reduced renal perfusion during AKI in COVID-19 are in line with prior observations in AKI due to bacterial septic shock [7, 9], and thoracic surgery [10]. Results of renal ultrasound in critical COVID-19 have also implied reduced perfusion either using contrast enhancement or as an indirect observation of larger values of resistance index [18, 19]. We also show increased resistive index correlated with lower total renal blood flow. Multiparametric MRI has previously been used in a study of nine patients with severe AKI of different etiologies at a median of six days after peak P-Creatinine, also demonstrating a reduced renal perfusion [17]. In our study, differences in renal perfusion between groups were partly attenuated after adjusting for total kidney volume (TKV). Reduced TKV (because of loss of functional mass) predisposes for AKI development, while AKI development in itself increases TKV [17]. Although we could not demonstrate significant differences in TKV between the groups, adjustment may have introduced more uncertainty to the data by these mechanisms.

A limitation to the above-mentioned studies using thermodilutional catheters [7, 10] or phase contrast MRI [9] to determine total renal blood flow is that regional hypoperfusion cannot be investigated. Using ASL MRI in this study, we additionally demonstrate reduced regional perfusion in both renal cortex and medulla.

Dehydration and reduced circulating blood volume resulting in hypoperfusion of the medulla is a well-known mechanism of AKI [20] and has been suggested as a major contributor to AKI development in severe COVID-19 [21]. However, the evident systemic inflammation with increased levels of cytokines in critical COVID-19 [22] is also associated with AKI development [23]. In animal experiments, systemic inflammation can cause AKI with normal kidney perfusion and even with hyperperfusion [24, 25]. As mean arterial pressure did not correlate with changes in regional perfusion in our study, renal autoregulation may still partly attenuate the consequences of hypoperfusion during normotensive conditions during critical COVID-19.

We find a discrepancy in the renal perfusion in our study when evaluated using phase contrast MRI compared to ASL, however, a strong correlation between values was shown (Fig. 3). Total perfusion assessed with PC-MRI was not indexed for renal or body size, whereas regional perfusion is expressed per 100 g functional tissue. Since the median TKV in both groups were similar, the relative difference between groups would be expected to be similar if the modalities were interchangeable. PC-MRI is sensitive to errors in planning the angle, whereas ASL-estimates depend on cortical mapping where inclusion of low-perfused areas in voxels reduces the estimated mean. RBF determined by phase contrast has a higher intra-individual variability than cortical ASL [15]. A similar discrepancy between perfusion between PC-MRI and ASL has been found in a previous study of CKD where the reduction in perfusion compared to healthy individuals was more pronounced using ASL compared to PC-MRI [15]. Therefore, values of these two modalities are not interchangeable, at least not during pathological conditions, and have qualitative differences. Taken together, they nonetheless strengthen the interpretation that renal perfusion is reduced early during AKI in ICU-patients with COVID-19.

Despite a marked reduction in regional perfusion in both cortex and medulla, we could not reveal differences in renal oxygenation in patients with AKI compared with those without, using either BOLD or TRUST sequences. In fact, BOLD imaging rather demonstrates the same level of renal oxygenation as healthy individuals of similar age. This is also similar to the findings when AKI patients were investigated 6 days after peak P-Creatinine [17]. A strength of TRUST is its insensitivity to hemodilution and edema. Renal venous saturation using TRUST in healthy volunteers has been estimated to 89 ± 2% by our group (unpublished data) which is close to values expected from measurements with renal vein catheters but differs from our study population [7]. As such, the TRUST-values here imply increased renal oxygen extraction in COVID-19 patients in general.

Our results do not support hypoperfusion-induced renal hypoxia as a specific feature of early AKI in COVID-19. Possible explanations as to how reduced perfusion in both medulla and cortex is not accompanied by detectable renal hypoxia include offsets in the relation between tpO_2_ and the BOLD signal during COVID-19-associated AKI. The BOLD signal is generated by the occurrence of deoxyhemoglobin, with a linear relationship between intrarenal deoxyhemoglobin content and *R*_2_* [26]. Increased water content decreases both *R*_2_ and *R*_2_* strongly and differences therein may attenuate differences in deoxyhemoglobin content. Influence of water content is supported by the correlations between *R*_2_*, *R*_2_, *T*_1_, and ADC in the patient group (Fig. 3). Further, intrarenal microthrombotization has also been demonstrated in COVID-19-associated AKI and may contribute to increased renal resistance [4, 27]. Since thrombotized vessels only transitorily contain deoxyhemoglobin, the effect on BOLD signal may not be detectable. Also, decreased intrarenal blood volume due to vasoconstriction or changes in oxygen transit in tissue could also offset the relation between tPO_2_ and renal oxygenation measured using BOLD [28]. We cannot conclude if these mechanisms contribute to our findings or to what extent.

Tissue composition and DWI parameters did not differ between the two patient groups but *T*_1_ values differed from healthy controls. We are unable to conclude if these findings are due to COVID-19 or caused by comorbidities. Previous investigations of patients with CKD found both lower ADC and longer *T*_1_ compared with healthy controls [16]. However, longer *T*_1_ is also found in the acute phase of AKI with a reduction to healthy population’s mean after a year of recovery accompanied with a reduction to normal of total kidney volume [17]. The higher *T*_1_ values may thus reflect higher water content in inflammatory, edematous tissue.

Both the early investigation and a comparator group of COVID-19 patients treated in the ICU without AKI add substance to the observations presented. Some limitations related to the MRI sequences have been addressed previously. Further limitations include that TRUST is a more novel sequence in renal MRI where pitfalls in the renal application is less explored. In our study, there were also more missing values due technical problems with this sequence and a larger variation in range of estimates in the TRUST measurements compared with BOLD. The COVID-19 cohort and the healthy volunteers imaging data were acquired on a different scanner, but importantly, this used the same sequences and we have reported similar measures between the two scanners in young healthy volunteers [11, 15]. As the main comparison is between patients with AKI and NO AKI with COVID-19, we do not consider this a major limitation. Relatively few patients have been included in both groups all from a single center. Patients in the AKI and the NO AKI differ besides renal function as patients in the AKI group were treated longer in the ICU, with higher proportion of IMV and with higher PEEP. We could not find a significant correlation between PEEP during the MRI examination and global or regional renal perfusion, but are unable to draw further conclusions regarding the influence of respiratory therapies. There is a skewness in the study population compared with ICU-populations with COVID-19 at large, since severely deteriorated patients where MRI was not feasible were excluded. Still, in our opinion, the disease severity of the cohort represents a relevant part of the patients in the ICU and the timing of the MRI examination in relation to the course of the disease represents a phase where therapeutic interventions are much needed.

## Conclusion

By using novel state-of-the-art techniques, this study demonstrates that in critically ill patients with COVID-19, patients with AKI have decreased total, cortical and medullary renal blood flow without effects on renal oxygenation compared with patients without AKI.

## Supplementary Information

**Additional file 1.** 1. Description of MRI data acquisition and Analysis.2. Scatterplots with correlation-lines and 95% confidence intervals of predicted mean of selected parameters from Fig. 3.

## Data Availability

The datasets generated and/or analyzed during the current study are not publicly available due national and EU regulations regarding patient related data, but are available from the corresponding author on reasonable request.

## Abbreviations

ACEi: Angiotensin converting enzyme inhibitor
ADC: Apparent diffusion coefficient
AKI: Acute Kidney Injury
ARB: Angiotensin II receptor blocking drug
ARDS: Acute respiratory distress syndrome
ASL: Arterial Spin Labeling
BOLD: Blood Oxygen Level Dependent
CKD: Chronic kidney disease
COPD: Chronic obstructive pulmonary disease
CRP: C-reactive protein
*D*: Pure diffusion
*D**: Pseudodiffusion
DWI: Diffusion weighted imaging
eGFR: Estimated glomerular filtration rate
eFF: Estimated filtration fraction
*Fp*: Perfusion fraction
ICU: Intensive Care Unit
IMV: Invasive mechanical ventilation
IQR: Interquartile range
KDIGO: Kidney Disease Improving Global Outcome
MAP: Mean arterial pressure
mpMRI: Multiparametric Magnetic Resonance Imaging
NT-proBNP: N-terminal pro-brain natriuretic peptide
PEEP: Positive end-expiratory pressure
P/f: PaO_2_/FiO_2_
PC: Phase contrast
*R*_2_*: BOLD relaxation rate
RBF: Renal blood flow
RRT: Renal replacement therapy
SAPS 3: Simplified Acute Physiology Score 3
SOFA: Sequential Organ Failure Assessment
*T*_1_: Longitudinal relaxation time
*T*_2_: Transverse relaxation time
TRUST: *T*_2_ Relaxation Under Spin Tagging
