# Supplementary material for: Decreased renal perfusion during acute kidney injury in critical COVID-19 assessed by magnetic resonance imaging: a prospective case control study
Source: Crit Care. 2022 Sep 1;26:262. doi: 10.1186/s13054-022-04132-8 (PMC9434518; doi:10.1186/s13054-022-04132-8)

Supplemental material for "Decreased perfusion during acute kidney injury in critical COVID-19 assessed by magnetic resonance imaging”

Index:

1. Description of MRI data acquisition and Analysis
2. Scatterplots with correlation-lines and 95% confidence intervals of predicted mean of selected parameters from Figure 3.
3. Description of MRI data acquisition and Analysis

*Data Acquisition*

Participants were scanned on a 3T MR scanner (Achieva dStream, Philips Healthcare, Best, The Netherlands). A 16-channel torso phased array coil together with a spine coil served for signal reception. Subjects were in supine position during scanning. The MRI protocol was designed to be ~ 35-40 minutes in duration, with MRI parameters guided by previous studies.(1-3).

Breath-hold balanced turbo field echo (bTFE) images were acquired in axial, sagittal and coronal directions to guide subsequent planning of scans. A T_2_-weighted turbo spin echo sequence (T_2_W TSE) was used to compute total kidney volume (TKV) (echo time (TE)/repetition time (TR) 908/60 ms, field of view (FOV) 350x350x104 mm^3^, in-plane resolution 1.5x1.5 mm^2^ and 5 mm slice thickness with 0.5 mm slice gap, 19 slices, bandwidth/pixel 792 Hz, breath hold duration 17s).

Phase contrast (PC) MRI data was applied to measure blood flow in each renal artery using a single slice ECG triggered turbo field echo (TFE) sequence (TE/TR 4.2/10.2 ms, reconstructed in-plane resolution 1.17x1.17 mm^2^ and 6 mm slice thickness, velocity encoding 120 cm/s, bandwidth/pixel 285 Hz). For each PC-MRI acquisition, 20 measures of blood flow across the cardiac cycle were collected during a single breath hold (15-20 sec).

Regional renal perfusion was quantified using a respiratory-triggered flow-sensitive alternating inversion recovery (FAIR) arterial spin labelling (ASL) sequence,(4) with selective/non-selective slab thickness of 45/400 mm. Data was collected at inversion times (TI) of 400, 600 and 1800 ms, with 5, 5 and 25 control/label image pairs collected at each TI, in addition an equilibrium magnetization (M_0_) image was collected. In the same space, a respiratory-triggered T_1_-mapping scheme (TIs: 200, 300, 400, 500, 600, 700, 800, 900, 1000, 1100, 1200, 1300 and 1500 ms) was collected.(1) Both the ASL and T_1_-mapping measures utilized a fat suppressed spin echo-echo planar imaging (SE-EPI) readout scheme (5 contiguous slices, TE 27 ms, slice acquisition order descend, temporal slice spacing 56ms, FOV 336x336x25 mm^3^, acquisition matrix 112x112, bandwidth/pixel 2200 Hz, spatial resolution 3x3x5 mm^3^) with readouts collected at the end of expiration.

A multiecho fast field echo (mFFE) sequence was used to quantify R_2_* = 1/T_2_*, defined as the blood oxygenation level dependent (BOLD) relaxation rate (TR 196 ms, FOV 336x336x25 mm^3^, 5 contiguous slices, slice thickness 5 mm, initial TE 5 ms, echo spacing 3 ms, 12 echoes, bandwidth/pixel 346 Hz, single breath hold ~12 s) with reconstructed resolution of 1.5x1.5x5 mm^3^. A respiratory triggered FAIR-based T_2_ Relaxation Under Spin Tagging (TRUST) scheme was collected in a sagittal slice through the left renal vein with effective TEs (eTE) of 1, 40, 80 and 160 ms, 6 selective/non-selective image pairs were acquired at each eTE at a PLD time of 1000 ms (FOV 244x244 mm^2^, in plane resolution 2.5x2.5 mm^2^).

Respiratory-triggered fat suppressed diffusion weighted imaging (DWI) was acquired with nine b-values of 0, 5, 15, 30, 45, 60, 75, 400 and 600 s/mm^2^ (SENSE 2.3, TE 49.7ms, FOV 336x336x24.5 mm^3^, matrix 112x112, in plane resolution 3x3 mm^2^, slice thickness/gap 4.5/0.5 mm).

T_2_ mapping was performed using a respiratory triggered gradient-echo spin-echo (GraSE) sequence (30 echoes of TE 12 – 186 ms in 6 ms steps, FOV 288x288x25 mm^3^, spatial resolution 3x3x5 mm).

*Data analysis*

TKV was computed by manually tracing the kidneys on the T_2_W TSE images excluding cysts not fully enveloped with kidney tissue (Medical Image Processing, Analysis and Visualization (MIPAV), version 8.0.2 (2018-02-13))(5). PC-MRI data of the renal arteries were analyzed using Segment software version 3.1 R8123 (<http://segment.heiberg.se>).(6) A region of interest (ROI) was drawn around the vessel wall and vessel tracking used for automatic edge detection and propagation through all phases across the cardiac cycle. Total renal blood flow (RBF) was then calculated as the mean flux of blood flow (ml/min) over the cardiac cycle in both renal arteries. Global perfusion to the kidneys was estimated by dividing total renal blood flow by TKV. Renal resistance index derived from the PC-MRI measured arterial velocity was determined bilaterally as (V_systole_-V_diastole_)/V_systole_ where V is the velocity of the blood flow in the renal artery.

ASL data were realigned to a base M_0_ image, individual perfusion weighted difference images (control-label) calculated and averaged to create a perfusion-weighted (ΔM) average image per TI. Tissue perfusion (f) maps (in ml/100 g tissue/min) were calculated using ΔM from the longest TI of 1800 ms (with maps generated both with and without an inflow time correction from the shorter TIs), T_1_ maps (see below), and M_0_ maps. (1) T_1b_ was assumed to be 1.55 s at 3 T,(7) whilst the blood-tissue partition coefficient λ was assumed to be 0.8 ml/g for kidney Perfusion maps with an inflow correction time were used to assess cortex perfusion, whereas those without were used to assess medullary perfusion. For T_1_ mapping, inversion recovery data was inspected for motion and discarded if necessary. The remaining data were fit on a voxel-by-voxel basis to a standard inversion recovery equation to generate a T_1_ map using in-house Matlab (The MathWorks, Inc) code.

mFFE data were fit voxelwise to form T_2_*/R_2_* maps from the exponential signal decay using in-house Matlab code. TRUST analysis was performed in Matlab, by subtracting each label from control at each eTE and averaging across repeats to generate the blood signal (ΔS(eTE)). Nine voxels with greatest intensities within the vessel were then averaged for each eTE and fit to ΔS(eTE)=S_0_ exp(eTE(1⁄T_1b_ -1⁄T_2b_ )) where S_0_ is the signal intensity of pure venous blood and the blood T_1_ (T_1b_) was assumed to compute blood T_2_ (T_2b_). Blood oxygen saturation was determined using a calibration curve with calculated T_2b_ and the individual hematocrit level. (8)

DWI data were fit to form apparent diffusion coefficient (ADC) maps (in mm^2^/s) by taking the log of the exponential signal decay. For the IVIM model, *D* was first fit for b-values of ≥200 s/mm^2^, assuming that the pseudodiffusion *D** component can be neglected above this value, and perfusion fraction *f_p_* was determined from the zero intercept of this fit. *D** was then computed from the mono-exponential fit using the precalculated values of *D* and *f_p_*.(9) GRaSE sequence T_2_ maps were computed online on the scanner.

To interpret the multiparametric maps, binary whole kidney masks were first formed by manual segmentation of the T_1_ and T_2_* map, and from these cortex and medulla were segmented. A histogram of T_1_ and T_2_* values across both kidneys was formed and peaks identified to form separate renal cortex and medulla masks (1). Cortex and medulla masks generated from the T_1_ histograms were applied to the T_1_, perfusion, ADC, D, D* maps, and those formed from the T_2_* map were applied to the R_2_* maps, manual segmentation of the cortex of the T_2_ maps was performed. The mode of each parameter was calculated for the right and left kidney, and the mean of this value computed across both kidneys.

1. Cox EF, Buchanan CE, Bradley CR, Prestwich B, Mahmoud H, Taal M, et al. Multiparametric Renal Magnetic Resonance Imaging: Validation, Interventions, and Alterations in Chronic Kidney Disease. Front Physiol. 2017;8:696.

2. Buchanan CE, Mahmoud H, Cox EF, McCulloch T, Prestwich BL, Taal MW, et al. Quantitative assessment of renal structural and functional changes in chronic kidney disease using multi-parametric magnetic resonance imaging. Nephrology, dialysis, transplantation : official publication of the European Dialysis and Transplant Association - European Renal Association. 2020;35(6):955-64.

3. Buchanan C, Mahmoud H, Cox E, Noble R, Prestwich B, Kasmi I, et al. Multiparametric MRI assessment of renal structure and function in acute kidney injury and renal recovery. Clinical Kidney Journal. 2021.

4. Kim SG, Tsekos NV. Perfusion imaging by a flow-sensitive alternating inversion recovery (FAIR) technique: application to functional brain imaging. Magn Reson Med. 1997;37(3):425-35.

5. McAuliffe MJ, Lalonde FM, McGarry D, Gandler W, Csaky K, Trus BL, editors. Medical Image Processing, Analysis and Visualization in clinical research. Proceedings 14th IEEE Symposium on Computer-Based Medical Systems CBMS 2001; 2001 26-27 July 2001.

6. Bidhult S, Hedstrom E, Carlsson M, Toger J, Steding-Ehrenborg K, Arheden H, et al. A new vessel segmentation algorithm for robust blood flow quantification from two-dimensional phase-contrast magnetic resonance images. Clin Physiol Funct Imaging. 2019;39(5):327-38.

7. Dobre MC, Ugurbil K, Marjanska M. Determination of blood longitudinal relaxation time (T1) at high magnetic field strengths. Magn Reson Imaging. 2007;25(5):733-5.

8. Lu H, Ge Y. Quantitative evaluation of oxygenation in venous vessels using T2-Relaxation-Under-Spin-Tagging MRI. Magn Reson Med. 2008;60(2):357-63.

9. Suo S, Lin N, Wang H, Zhang L, Wang R, Zhang S, et al. Intravoxel incoherent motion diffusion-weighted MR imaging of breast cancer at 3.0 tesla: Comparison of different curve-fitting methods. J Magn Reson Imaging. 2015;42(2):362-70.

2. Scatterplots with correlation-lines and 95% confidence intervals of predicted mean of selected parameters from Figure 3, in 19 patients with and without AKI treated in intensive care due to COVID-19 and examined with renal multiparametric MRI. In:

a) the relation between resistive index in the renal arteries and total renal blood flow, both assessed with phase contrast MRI,

b) the correlation between T_2_ relaxation time and renal oxygenation (R_2_*) in Cortex,

c) the correlation between apparent water diffusion (ADC) using diffusion weighted MRI and renal oxygenation (R_2_*) in Cortex

d) the correlation between apparent water diffusion (ADC) using diffusion weighted MRI and T_1_ relaxation time in Cortex

e) the correlation between apparent water diffusion (ADC) using diffusion weighted MRI T_2_ relaxation time in Cortex

are presented.


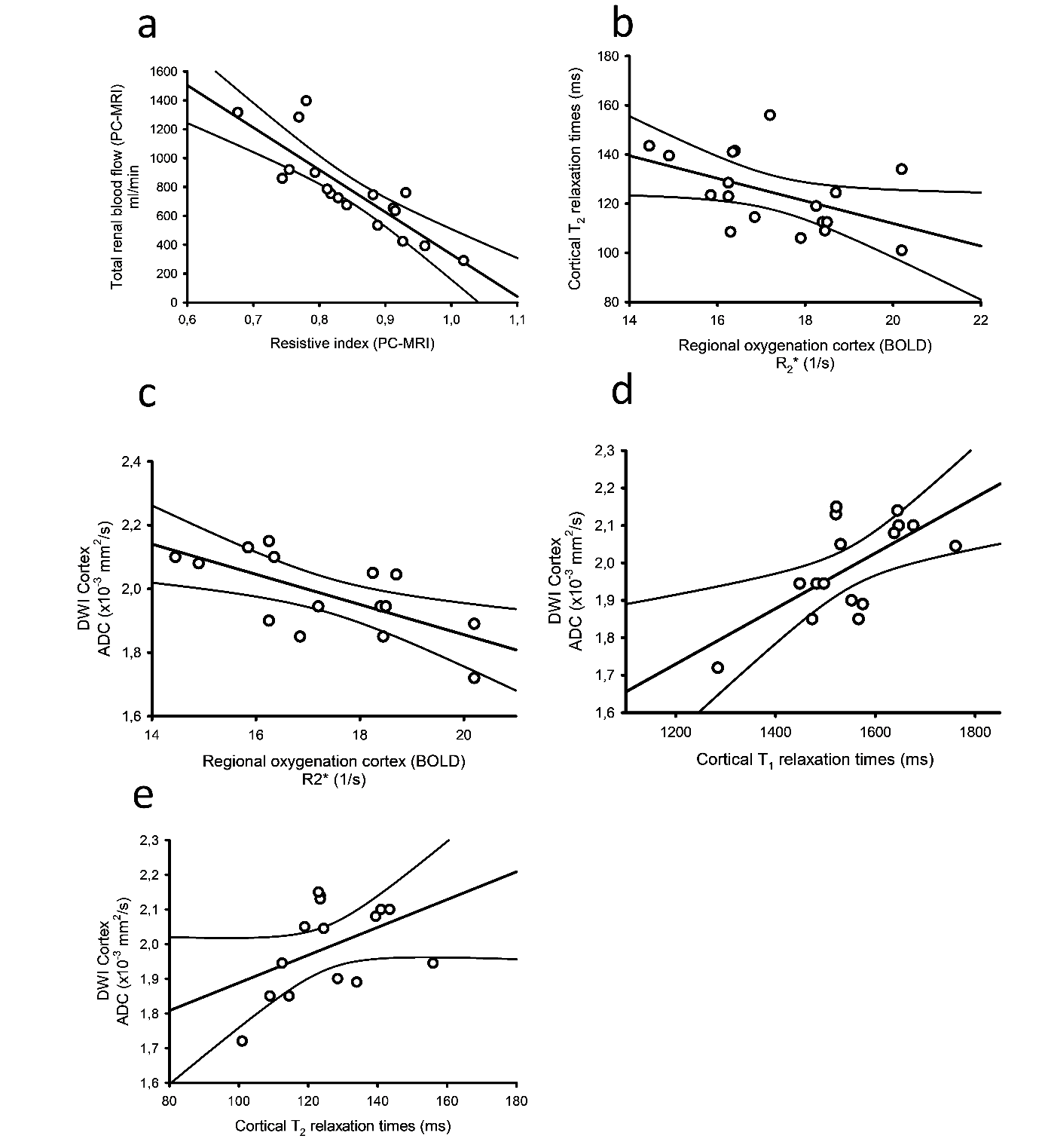

Supplement: Supplementary file 1 — Additional file 1. 1. Description of MRI data acquisition and Analysis.2. Scatterplots with correlation-lines and 95% confidence intervals of predicted mean of selected parameters from Fig. 3. [file 13054_2022_4132_MOESM1_ESM.docx]
